# Synthesis and evaluation of polyamine carbon quantum dots (CQDs) in *Litopenaeus vannamei* as a therapeutic agent against WSSV

**DOI:** 10.1038/s41598-020-64325-5

**Published:** 2020-04-30

**Authors:** Huai-Ting Huang, Han-Jia Lin, Hui-Ju Huang, Chih-Ching Huang, John Han-You Lin, Li-Li Chen

**Affiliations:** 1 Department of Aquaculture, National Taiwan Ocean University, No. 2, Pei-Ning Road, Keelung, 20224 Taiwan, ROC; 2 Department of Bioscience and Biotechnology, National Taiwan Ocean University, No. 2, Pei-Ning Road, Keelung, 20224 Taiwan, ROC; 3 Institute of Marine Biology, National Taiwan Ocean University, No. 2, Pei-Ning Road, Keelung, 20224 Taiwan, ROC; 4 Institute of Biological Chemistry, Academia Sinica, No. 128, Academia Road Sec. 2, Nankang, Taipei 115 Taiwan, ROC; 5 School of Veterinary Medicine, National Taiwan University, No. 1, Sec. 4, Roosevelt Rd., Taipei, 10617 Taiwan, ROC; 6 Centre of Excellence for the Oceans, National Taiwan Ocean University, No. 2, Pei-Ning Road, Keelung, 20224 Taiwan, ROC

**Keywords:** Virus-host interactions, Nanoparticles

## Abstract

White spot syndrome virus (WSSV) is the causative agent of white spot syndrome (WSS), a disease that has led to severe mortality rates in cultured shrimp all over the world. The WSSV is a large, ellipsoid, enveloped double-stranded DNA virus with a wide host range among crustaceans. Currently, the main antiviral method is to block the receptor of the host cell membrane using recombinant viral proteins or virus antiserum. In addition to interference with the ligand-receptor binding, disrupting the structure of the virus envelope may also be a means to combat the viral infection. Carbon quantum dots (CQDs) are carbonaceous nanoparticles that have many advantageous characteristics, including small size, low cytotoxicity, cheap, and ease of production and modification. Polyamine-modified CQDs (polyamine CQDs) with strong antibacterial ability have been identified, previously. In this study, polyamine CQDs are shown to attach to the WSSV envelope and inhibit the virus infection, with a dose-dependent effect. The results also show that polyamine CQDs can upregulate several immune genes in shrimp and reduce the mortality upon WSSV infection. This is first study to identify that polyamine CQDs could against the virus. These results, indeed, provide a direction to develop effective antiviral strategies or therapeutic methods using polyamine CQDs in aquaculture.

## Introduction

Several pathogens cause illness even death in shrimp. Among them, the white spot syndrome virus (WSSV) is a serious disease and causes severe economic losses on shrimp culture industry^1–4^. WSSV infects all decapod crustaceans and has the strongest infectivity and lethality in penaeid shrimp, including *Penaeus monodon*, *Litopenaeus vannamei*, *P. chinensis* and *Marsupenaeus japonicus*. When the penaeid shrimp were infected with WSSV, the mortality rate can reach 100% within 2–7 days^4,5^. This virus was first detected in Taiwan in 1992^6^ and subsequently was found in Japan and other Asian countries, as well as South Texas, Ecuador and Brazil^7,8^. Because of the broad host range, strong infectivity and high mortality, WSSV is very difficult to prevent and control. The WSSV is an enveloped virus, and some virus viral particles possess a tail-like projection at one end. They are about 210–420 nm in length and about 70–167 nm in diameter^8,9^. Because shrimp have an open circulatory system, the virus can easily spread everywhere in the body via the haemolymph^10^. WSSV mainly infect the ectodermal organs (the epidermis, the former intestine, hindgut, gill and nerve tissue) and mesodermal organs (lymphatic organs, antennal gland, connective tissue and hematopoietic tissue) in shrimp, but are not usually found in the endodermal organs (hepatic, pancreatic ductal and midgut epithelium)^5,11,12^.

Although the mechanism of virus entry is not fully understood, several proteins and receptors have been found to participate in WSSV infection^13,14^. The virus first interacts with cell membrane proteins to enter into the host cell by endocytosis and starts to replicate; thus, the interaction between the host receptor and the virus’ ligand is important^13^. The main antiviral methods currently used block the receptor of host cell membrane using recombinant viral proteins or virus antiserum. WSSV can be neutralized by the VP24, VP28, VP26 and VP19 antisera^13,15–19^; with the VP28 antiserum significantly inhibiting mortality during infection^19^. There are also many recombinant proteins, like VP26, VP28, VP39, VP51B, VP53A, chitin binding protein (CBP), and glucose transporter 1 (Glut1), that can inhibit WSSV infection^14,20^. In addition to interfering with the ligand-receptor binding relationship, disrupting the structure of the virus envelope may also be a means of combating viral infection. However, the materials capable of destroying the virus envelope to inhibit virus infection are scarce, and there are no commercial treatments available to treat or prevent the WSSV infection until now.

Carbon quantum dots (CQDs) are carbon-based nanoparticles which can be produced and modified easily and have desirable characteristics, such as small size and low cytotoxicity^21,22^. In addition, CQDs exhibit antibacterial ability by inducing oxidative stress through the production of reactive oxygen species (ROS)^23,24^. Besides, carbon nanoparticles have been verified to be safer than metal nanoparticles by our team members^25^ and other research teams. Polyamines can bind with DNA, lipids, and proteins, which are involved in many important mechanisms like cell growth, proliferation, and death^26^. Polyamine-modified CQDs (polyamine CQDs) have a diameter of 6 nm and high solubility^27^. Polyamine CQDs have a strong antibacterial efficiency, as their highly positive charge severely disrupt the bacterial membrane. Previous studies confirm that polyamine CQDs are effective against *Escherichia coli*, *Staphylococcus aureus*, *Bacillus subtilis*, and *Pseudomonas aeruginosa*^26^. Many nanoparticles, such as silver and gold nanoparticles, have been used as treatments against viruses^28–30^. Therefore, safe and cheap polyamine CQDs could be a potential anti-bacterial material to replace antibiotic in aquaculture. In addition, the WSSV is an enveloped virus, so polyamine CQDs may destroy the viral envelope to affect the invasion of the virus.

Shrimp are invertebrates depend on the innate immunity to avoid the adverse effects of pathogens^31^. The innate immune mechanisms mount non-specific immune responses which are immediate but do not induce defence responses against pathogenic invasion. There are several innate immune responses in shrimp, including the prophenoloxidase (proPO) system, haemolymph clotting mechanism, antioxidant defence mechanism, antimicrobial immune responses and pattern recognition receptors^31,32^. Some research has indicated that nanoparticles are immunostimulatory and immunosuppresive abilities. ZnO and silver nanoparticles (AgNPs) could induce the innate immune response in *Penaeus semisulcatus* and *L. vannamei*^33,34^. The AgNPs contained diet could help shrimp to against WSSV, *Vibrio cholerae, V. harveyi*, and *V. parahaemolyticus* infection^33,35,36^. Gold nanoparticles (AuNP) has immunostimulatory effects to against the infection of *V. parahaemolyticus*^37^. Therefore, this study also explores the ability of polyamine CQDs to regulate the immune system of shrimp.

## Materials and Methods

### Experimental animals

The *Litopenaeus vannamei* shrimp used in this study were obtained from Kaohsiung, Taiwan. The shrimp were collected and acclimatized in the laboratory for 1 week before starting experimentation and confirmed to be WSSV-free by using the IQ2000™ WSSV diagnostic kit (GeneReach Biotechnology Corp.) (data not show). Throughout the acclimatization and experimental period, shrimp were fed twice daily with commercial shrimp feed, with or without the addition of polyamine CQDs. The temperature and salinity conditions of water were maintained at 25 ± 1 °C and at 34 ± 1‰.

### WSSV purification

The WSSV strain was collected from WSSV-infected *Penaeus monodon*, in 1994, in Taiwan^11^. The haemolymph of these shrimps were collected with phosphate‐buffered saline and stored at −80 °C. These WSSV solutions were injected into healthy crayfish *Procambarus clarkii*, and the dying crayfish were collected 7 days post-injection. The virions were purified from the crayfish according to the methods of Lee and Chen^20^. The virion concentration was calculated according to the method described by Zhou *et al*.^38^.

### Polyamine CQDs preparation

Polyamine CQDs were obtained from Dr. Chih-Ching Huang’s lab, National Taiwan Ocean University. The preparation has been described in a previous study^27^.

### Primary haemocyte culture and virus infection

Haemolymph were collected from the shrimp *Litopenaeus vannamei* using a syringe containing cold anti-coagulant (27 mM sodium citrate, 336 mM NaCl, 115 mM glucose and 9 mM EDTA, pH 7.0). The haemocytes were counted and seeded in 24 well plates (5 × 10^5^ cell/well). After seeding at room temperature for 30 min, the supernatants were removed. The attached cells were washed three times with 2× Leibovitz’s L-15 medium (Invitrogen). The cells were cultured in 500 μL 2× Leibovitz’s L-15 containing 20% foetal bovine serum (FBS), 2 g/L glucose, 100 UI/mL penicillin, 100 μg/mL streptomycin and 2.4% NaCl. WSSV (10^6^ copies) were incubated in 1.5 mL tubes with 20 ppm polyamine CQDs and 1 ppm polyamine CQDs, for 30 min, at room temperature. The mixtures of polyamine CQDs and WSSV were then incubated with the haemocytes, at room temperature, in a 24-well microplate. After incubation for 6 h, the haemocytes were washed with PBS and the real-time PCR procedure was conducted.

### RNA extraction and reverse transcription-PCR

Total RNA was isolated using TRIzol reagent (Invitrogen), according to manufacturer’s instructions. The primary haemocytes (5 × 10^5^ cell per well), and shrimp haemocytes for later experiments, were lysed with 300 μL TRIzol reagent. The mixture was precipitated using 2‐propanol and ethanol. Total RNA was centrifuged in 75% ethanol at 12,000 g, at 4 °C, for 5 min. The pellets were resuspended in diethyl pyrocarbonate (DEPC) water and quantified by spectrophotometry. After RNA extraction, 1 μg aliquot of RNA was treated with 1 U of RNase-free deoxyribonuclease I (DNase I) (Thermo Fisher Scientific) at 25 °C, for 30 min, to remove genomic DNA contamination and then EDTA was added. The DNase‐treated total RNA was denatured by heating at 65 °C for 5 min in 10 μL DEPC water, containing 50 μM oligo dT‐anchor primer (Roche) and 1 μL of 10 μM dNTP mixture. The first‐strand cDNA was synthesised by the addition of 5 μL of 10× First-Strand buffer, 2 μL 0.1 M DTT, 1 μL 40 U RNase inhibitor (Thermo Fisher Scientific) and 1 μL HiScript I reverse transcriptase (Bionovas). The reaction proceeded at 42 °C for 60 min and was terminated by heating at 75 °C for 15 min. Then, the cDNA was treated with 1 mL RNase H to remove the RNA template from the DNA.

### Real-time PCR analysis

The primers for real-time PCR used in this study are according to Wang *et al*.^39^. Real-time PCR was conducted using the RealQ Plus 2× Master Mix Green, Low ROX (Ampliqon) and performed using the ABI 7500 Fast Real-Time PCR System (Applied Biosystems). For WSSV *immediate early 1* (*ie1*) gene, the forward primer 5′-TGACCCACTCCATGGCCTT-3′ and the reverse 5′-CAAGTACCCAGGCCCAGTGT-3′ were used. For the *Elongation factor 1-α* (*EF1-α*) gene, the forward primer 5′-GCCAGGTATGGTTGTCAACTTTG-3′ and the reverse 5′-GCCACGCTTCAGATCCTTCA were used. The analysis was conducted according to Livak and Schmittgen^40^.

### Cryo-electron microscopy (cryo-EM)

Four μL of each prepared sample was deposited onto a 300-mesh holy carbon film grid (HC300-Cu) which was glow-discharged at 20 mA for 15 s. The grid was flash frozen in liquid ethane using a Vitrobot (FEI). All specimens were imaged on a Tecnai F20 Twin transmission electron microscope operating at 200 keV, using an electron dose of 20 e /Å2, on a Gatan 4k x 4k CCD, with a magnification of 50,000x and defocus values ranging from 1500 to 2000 nm.

### Preparation of test diets

Polyamine CQDs were dissolved in the water to the required concentration. For coating, the polyamine CQDs solution was mixed with the feed and incubated at room temperature for 15 min. This allowed the polyamine CQDs to be absorbed into the feed and prevented its dispersion in water. The feed was then dried and stored at room temperature until use.

### *In vivo* experiments

For the immune gene analysis, the experimental shrimp were divided into 3 groups containing three shrimps each, and with three replicates of each group. The shrimp were provided with the following treatments: group 1 with feed containing 10 ppm polyamine CQDs, group 2 with feed containing 1 ppm polyamine CQDs, and group 3 with feed containing PBS, as the negative control. During the experimental period, shrimp were fed twice daily with commercial shrimp feed or polyamine CQDs-containing feed. Water was kept at 25 ± 1 °C and a salinity of 34‰. After 7 days, the haemolymph was collected from the shrimp, for the real-time PCR procedure.

For virus infection, the experimental shrimp were divided into 3 groups, with three replicates of 20 shrimps in each group. The natural transmission routes of virus in shrimp aquatic farming are feeding and immersion^41^. Thus, in this study challenging shrimp by feeding with infected shrimp meat was applied to monitor the natural infection. The shrimp were treated as follows: group 1 was challenged with WSSV using infected shrimp meat, as the positive control, group 2 was challenged with WSSV using infected shrimp meat, plus 10 ppm polyamine CQDs, and group 3 with PBS, as the negative control. For group 1 or group 2, the shrimp were fed with commercial shrimp feed or 10 ppm polyamine CQDs-containing feed for 7 days, and then infected with WSSV using infected shrimp meat. During the acclimatization and experimental period, shrimp were fed twice daily with commercial shrimp feed or polyamine CQDs-containing feed. Water conditions were maintained at 25 ± 1 °C and a salinity of 34‰. Shrimp mortality was recorded twice, daily. The mortality rates were subjected to paired sample t-tests.

## Result

### *In vitro* inhibition of WSSV infection by polyamine CQDs

**Figure 1:**
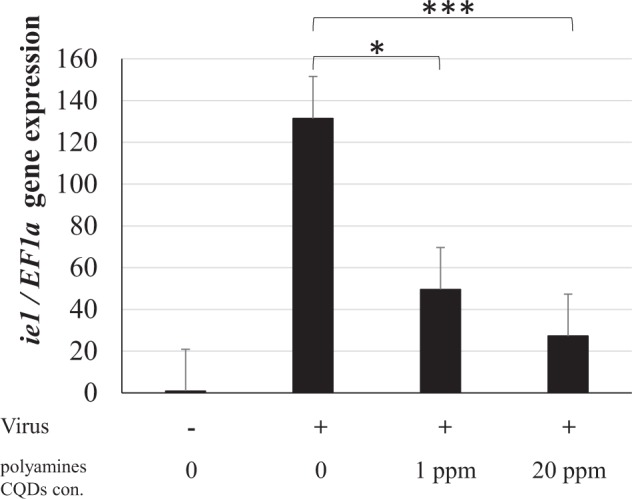
Identification of polyamine CQD anti-viral ability *in vitro*, by real-time PCR. The virus was treated with 20 or 1 ppm polyamine CQDs, and infected haemocytes. The expression of *ie1* was used as a WSSV infection indicator and *EF1-α* was set as control. The experiment was repeated three times. Each bar represents the mean ± SD. The statistical significance was calculated using Student’s *t*-test. Significant differences (*p* < 0.05) in gene expression level between the compared groups are indicated with two asterisks. Triple asterisks indicated greater significant difference (*p* < 0.0005).

The experiments using primary cultured haemocytes were performed in order to determine whether polyamine CQDs could inhibit WSSV infection. The virus was pretreated with 20 or 1 ppm polyamine CQDs for 1 hour, and then co-cultured with the primary cultured haemocytes for 6 hours. The intensity of WSSV infection were determined by real-time PCR. The WSSV immediate early gene (*ie1*) is a viral transcription factor, so it could be detected at early infection^42^. *Elongation factor 1-α* (*EF1-α*) gene was set as the internal control^43^. Figure 1 shows that WSSV pretreated with polyamine CQDs had low-level virus infectivity. Even if no significant differences were found between 20 ppm group and 1 ppm group, polyamine CQDs do decrease virus infection *in vitro* test.

### Polyamine CQDs could attach to WSSV envelope

**Figure 2:**
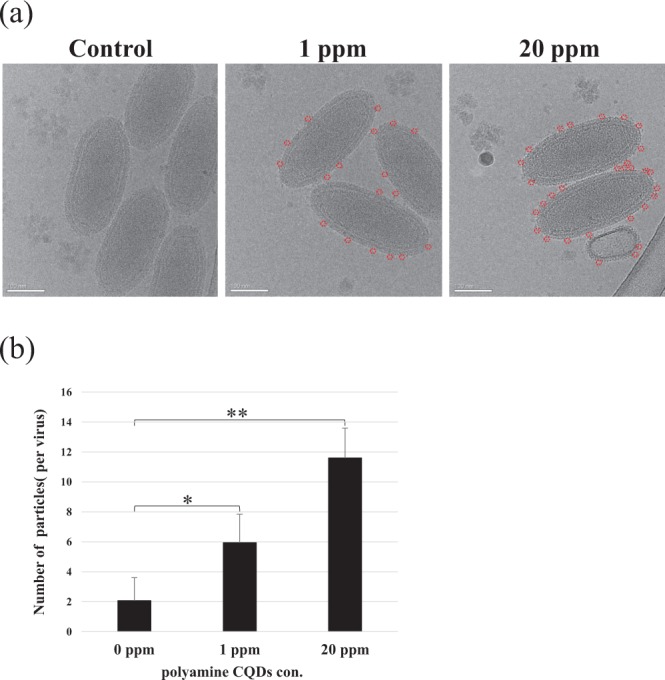
Cellular localizations of polyamine CQDs in WSSV by cryo-EM. (**a**) The virus treated with PBS (0 ppm, control), 1 or 20 ppm polyamine CQD. Red circles are polyamine CQDs. Scale bar: 100 nm. (**b**) Comparison of the number of polyamine CQDs around control and polyamine CQDs-containing virus. The statistical significance was calculated using Student’s *t*-test. Significant differences (*p* < 0.05) between the compared groups are indicated with asterisk. Double asterisks indicate very significant differences (*p* < 0.005).

To explore the antiviral mechanism of polyamine CQDs, the viruses, treated with 20 ppm and 1 ppm polyamine CQDs, were observed by microscope. Cryo-electron microscopy (cryo-EM) is used to observe the polyamine CQDs because polyamine CQDs could not recognize by any antibody and conjugate with fluorescence. Due to their size, polyamine CQDs appear as very small, dark spots under the cryo-EM^27^. Between 6 and 11 such dots were attached to the envelope of one polyamine CQDs-treated virus (Fig. 2a), which was higher than the control group (Fig. 2b).

### Shrimp immune gene expression after being fed polyamine CQDs

In order to understand the innate immune system of shrimp in applications of polyamine CQDs in shrimp aquatic farming, oral feed is applied. Real-time PCR was performed to analyze the gene of shrimp immune systems after treatment with the polyamine CQDs-containing feed.

### proPO activating system

**Figure 3:**
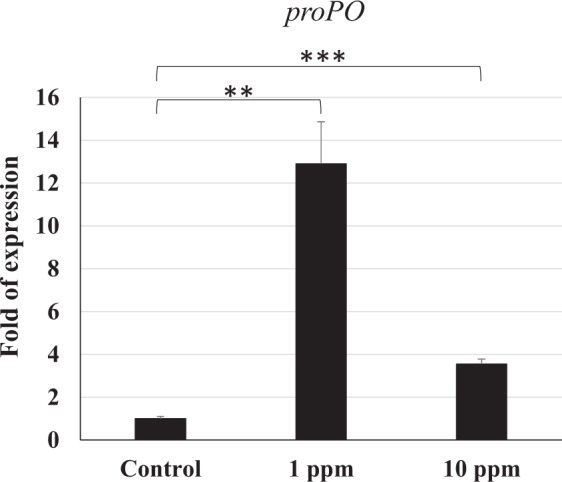
The gene expressions of *proPO* in shrimp haemocyte after feeding polyamine CQD containing-feed, evaluated by real-time PCR. The experiment was repeated three times. Each bar represents the mean ± SD. The statistical significance was calculated using Student’s *t*-test. Very significant differences (*p* < 0.005) of gene expression level between the compared groups are indicated with double asterisks. Triple asterisks indicated greater significant difference (*p* < 0.0005).

The results showed that the level of *prophenoloxidase (proPO)* gene expression in shrimp treated with the 10 ppm polyamine CQDs-containing feed group was not higher than the 1 ppm polyamine CQDs-containing feed group. However, the gene was upregulated in shrimp that received the polyamine CQDs-containing feed (Fig. 3).

### Haemolymph clotting mechanism

**Figure 4:**
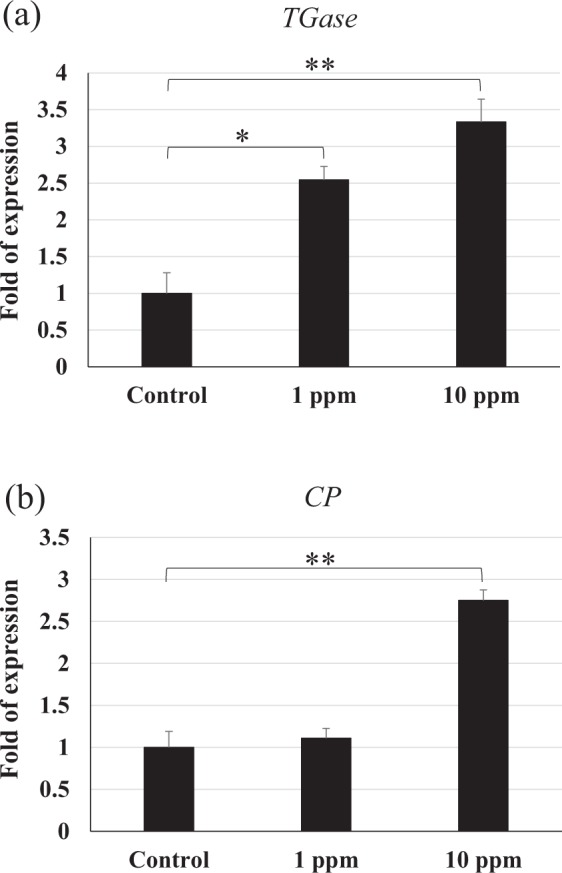
The relative gene expressions of haemolymph clotting mechanism in shrimp haemocyte after feeding polyamine CQD containing-feed, evaluated by real-time PCR. (**a**) *TGase* and (**b**) *CP* gene expressions in shrimp haemocyte. The experiment was repeated three times. Each bar represents the mean ± SD. The statistical significance was calculated using Student’s *t*-test. Significant differences (*p* < 0.05) of gene expression level between the compared groups are indicated with double asterisks. The double asterisks indicated greater significant difference (*p* < 0.005).

The results in Fig. 4a show that *transglutaminase (TGase)* gene was upregulated in shrimp that were fed the polyamine CQDs-containing feed. This expression was higher in the 10 ppm polyamine CQDs-containing feed group compared to the 1 ppm polyamine CQDs-containing feed group. *Clotting protein (CP)* gene expression was no different after in the 1 ppm polyamine CQDs-containing feed group, but the gene was upregulated in the 10 ppm polyamine CQDs-containing feed group (Fig. 4b).

### Antimicrobial peptide system

**Figure 5:**
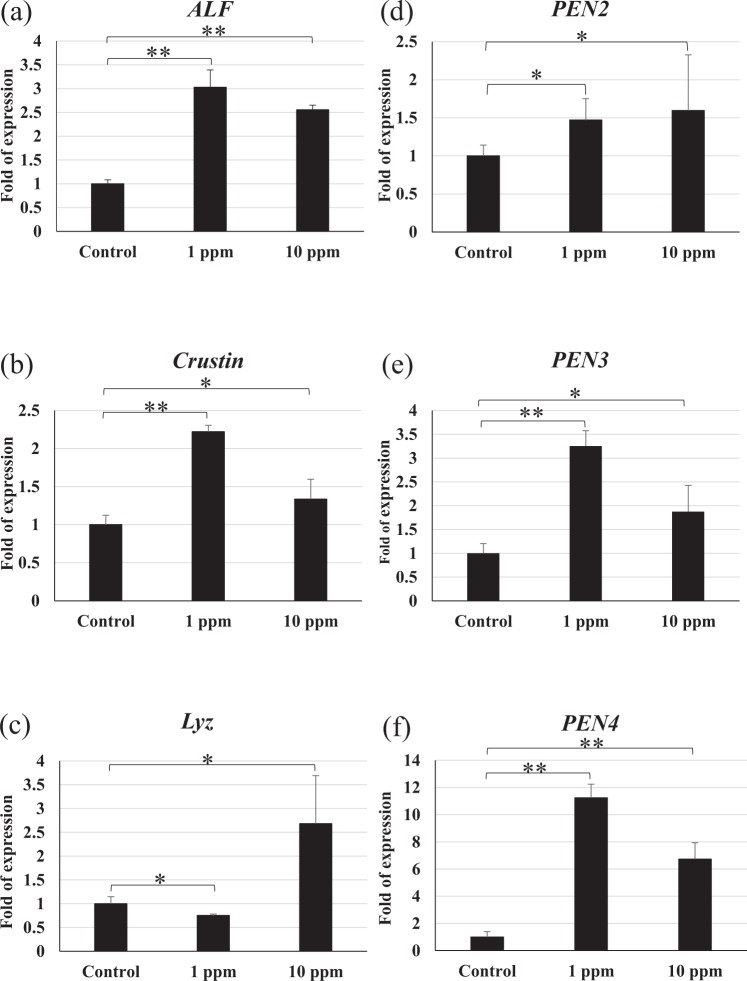
The relative gene expressions of antimicrobial peptide system in shrimp haemocyte after feeding polyamine CQD containing-feed, evaluated by real-time PCR. (**a**) *ALF*, (**b**) *Crustin*, (**c**) *Lyz*, (**d**) *PEN2*, (**e**) *PEN3* and (**f**) *PEN4* gene expressions in shrimp haemocyte. The experiment was repeated three times. Each bar represents the mean ± SD. Significant differences (*p* < 0.05) of gene expression level between the compared groups are indicated with one asterisk. Double asterisks indicate very significant difference (*p* < 0.005).

*S*ix of these antimicrobial peptide genes, *anti-lipopolysaccharide factor* (*ALF*), *crustin*, *lysozyme* (*Lyz*), *penaeidin 2* (*PEN2*), *penaeidin 3* (*PEN3*) and *penaeidin 4* (*PEN4*) were induced in shrimp that were fed polyamine CQDs-containing feed (Fig. 5).

### Antioxidant defence mechanism

**Figure 6:**
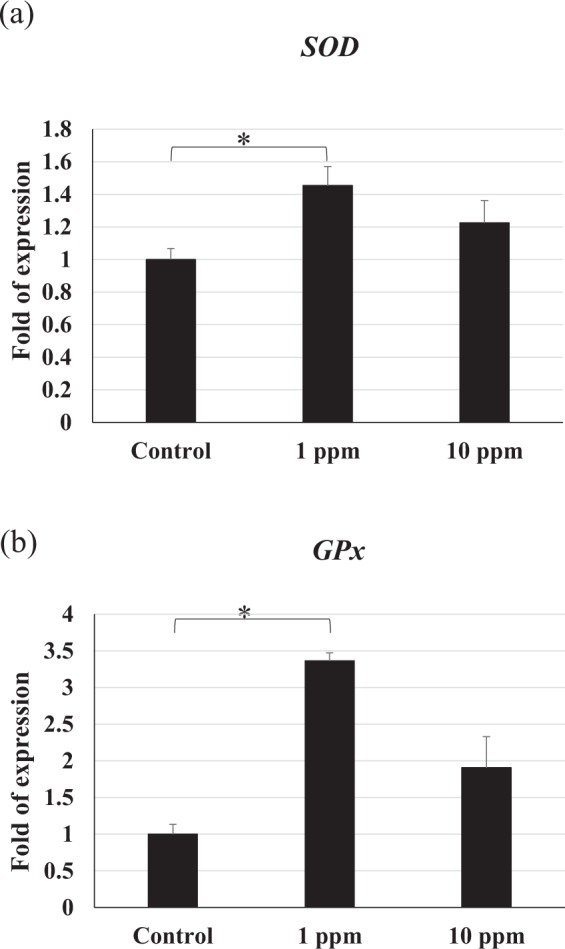
The relative gene expressions of antioxidant defence mechanism in shrimp haemocyte after feeding polyamine CQD containing-feed, evaluated by real-time PCR. (**a**) *SOD* and (**b**) *GPx* gene expressions in shrimp haemocyte. The experiment was repeated three times. Each bar represents the mean ± SD. Significant differences (*p* < 0.05) of gene expression level between the compared groups are indicated with one asterisk.

*Superoxide dismutase (SOD)* and *glutathione peroxidase (GPx)* gene expression were not significantly affected after treatment with 10 ppm polyamine CQDs-containing feed, but it was upregulated in shrimp after treatment with 1 ppm polyamine CQDs-containing feed. (Fig. 6).

### Pattern recognition receptors

**Figure 7:**
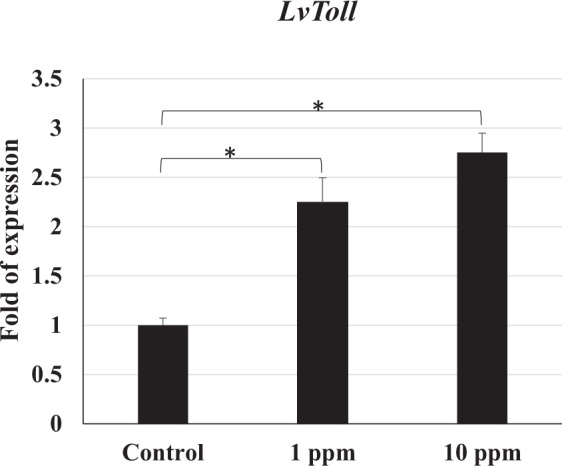
The gene expressions of *LvToll* in shrimp haemocyte after feeding polyamine CQD contained-feed, evaluated by real-time PCR. The experiment was repeated three times. Each bar represents the mean ± SD. Significant differences (*p* < 0.05) of gene expression level between the compared groups are indicated with one asterisk.

In this study, the results showed that the *LvToll* gene in shrimp was upregulated after being fed the polyamine CQDs-containing feed, and the gene expression level in the 10 ppm polyamine CQDs-containing feed group was higher than in the 1 ppm polyamine CQDs-containing feed group (Fig. 7).

### Assessment of antiviral activity *in vivo*

**Figure 8:**
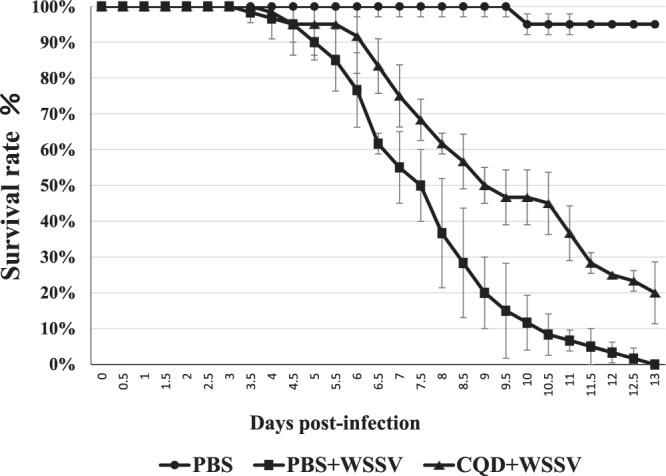
Identification of CQD anti-viral ability *in vivo*. The experiment was done using 20 shrimps in each group and repeated 3 times. Each bar represents the mean ± SD.

Since feed containing 10 ppm polyamine CQDs could induce many immune genes in shrimp, this concentration was used. The result shows that the mortality rate in the group treated with the polyamine CQDs-containing feed was much lower than the control group, implying that polyamine CQDs do actually play an important role in WSSV infection (Fig. 8).

## Discussion

Viral diseases are often the most difficult to treat. Most antiviral drugs inhibit viral functions such as genome duplication. Unlike most antibiotics, antiviral drugs do not destroy their target pathogen; instead they inhibit their development. This will cause damage to the hosts, and the virus is also prone to antiviral drug resistance. Therefore, in the face of viral diseases, researchers will eventually hope to find a safe, simple and cheap anti-virus strategy. In this study, we found that polyamine CQDs may be a candidate for expectations.

Many nanoparticles have been used as treatments against viruses. With reference to previous research, silver nanoparticles (AgNPs) inhibit hepatitis B virus (HBV)^29^, herpes simplex virus type 2 (HSV-2)^30^ and human immunodeficiency virus 1 (HIV-1)^44,45^ in vertebrate and against WSSV, *Vibrio cholerae*, *V. harveyi*, and *V. parahaemolyticus* infection in shrimp^33,35,36,46^. Gold nanoparticles (AuNP) reduce the replication of foot-and-mouth disease virus (FMDV) in cloven-hoofed animals^47^ and against the infection of *V. parahaemolyticus* in shrimp^37^. Titanium dioxide (TiO_2_) can deactivate rotavirus, astrovirus, and feline calicivirus (FCV)^48^. Our pervious study confirmed that polyamine CQDs decrease eye-related bacterial infections in rabbits. In this study, polyamine CQDs could fight against WSSV infection. The results have shown that polyamine CQDs can inhibit virus infection and have a dose-dependent effect. These results were also confirmed through *in vivo* experiments, which showed that shrimp administered polyamine CQDs-containing feed had a high survival rate. Peptide-nanoparticles conjugate the influenza A virus and inhibit binding of the influenza A virus to the host^49^. The sulfonate-capped silver and gold nanoparticles attach to herpes simplex virus type 2 (HSV-2) to inhibit infections^28^. Cryo-EM results showed that many polyamine CQDs attach themselves around the virus envelope (Fig. 2). Although the current information does not explain the correct mechanism, the anti-virus strategy of polyamine CQDs can be speculated that polyamine CQDs attach themselves to the virus to destroy the envelop structure, or to block the entrance of the virus, preventing infection of the cells. This must be done after further experiments.

Many nanoparticles have immune regulatory functions in invertebrates. *Mytilus galloprovincialis* haemocytes rapidly take up different types of nanoparticles to affect phagocytic function, and these particles also induce pro-apoptotic processes^50–52^. TiO_2_ bioaccumulates in earthworm tissue and induces many mechanisms, including decreased phagocytosis, increased superoxide dismutase mRNA, and apoptosis^53^. Nano-silver stimulates the SOD reaction in the organism. Recently, some research indicated that many nanoparticles have been added to feed to help shrimp against pathogens. 2 ppm AuNP containing feed that enhance *TLRs3* and *proPO* gene expression in *Litopenaeus vannamei* increased resistance to *V. parahaemolyticus* infection^37^. The 10 ppm biosynthesized AgNPs containing feed could induce immune gene to decrease *V. parahaemolyticus* infection in *L. vannamei*^33^. *L. vannamei* postlarval feed with 4 ppm colloidal silver have high mortality after challenged with *V. parahaemolyticus*^54^. In this study, shrimp fed with polyamine CQDs were protected against WSSV challenge. Thus, the impacts of oral treatment with polyamine CQDs in immune system were further analyzed.

The shrimp is an invertebrate species, for whom the innate immune system is very important. The prophenoloxidase (proPO) system is regarded as a major innate defense system of shrimp. The proPO protein is synthesized and stored in haemocytes, and then secreted into the haemolymph. It can against pathogens by triggering the downstream reaction, including melanisation, cytotoxic reactions, cellular adhesion, encapsulation and phagocytosis^31^. Figure 3 show that polyamine CQDs could induce *proPO* gene expression. In addition, pattern recognition receptors (PRRs) recognize pathogens and trigger downstream reactions against them. There are 11 PRRs that have been found in shrimp, including the Toll receptor^55^. *LvToll* 1 is an important innate immune gene for defending against *V. harveyi* infection^39^. *TLR3* gene expression of shrimp fed with 0.2–20 ppm AuNP show a dose-dependent upregulation at 24 h posttreatment^37^. Figure 7 indicated that 10 ppm polyamine CQDs could improve the *Toll* gene expression of shrimp when compared to 1 ppm and control group. However, downstream immune genes have to be analyzed to confirm the effect of polyamine CQDs in shrimp immune system.

In this study, the results indicated that polyamine CQDs stimulated many innate downstream immune responses, including haemolymph clotting mechanism, antioxidant defence mechanism, antimicrobial peptide system, and antimicrobial immune responses. The clotting system is the first line of defence against pathogens. It responds quickly to prevent haemolymph loss during injury and promotes wound healing^56^. TGase is released from the haemocytes after foreign particle stimulation or tissue damage, and the activated TGase triggers the polymerisation of the clotting protein (CP)^57^. Phagocytosis of haemocytes is an important defence mechanism in invertebrates^58^. After phagocytosis, reactive oxygen intermediates (ROIs) that exhibit cell toxicity are generated against the pathogens; however, these ROIs also damage the neighbouring cells. Therefore, the host produces some antioxidant enzymes, like superoxide dismutase (SOD) and glutathione peroxidase (GPx), to neutralize the ROIs^59^. Polyamine CQDs could also induce SOD reactions, with the exception of the GPx gene in the 10 ppm polyamine CQDs-treated feed group. This could be due to the fact that the dose was too high for the shrimp. Antimicrobial peptides (AMPs) are powerful antibacterial proteins, and several AMPs have been found in shrimp, including ALF, Crustin and Penaeidins^60^. ALF neutralises lipopolysaccharides and has strong antibacterial activity against Gram-negative bacteria^60^. Crustin is an antimicrobial peptide that has been found in various crustaceans. It is more effective against Gram-positive bacteria, compared to Gram-negative bacteria^61^. Penaeidins are a class of antimicrobial peptides that only exist in the family Penaeidae. There are 5 Penaeidin subfamilies that have been identified: PEN1, PEN2, PEN3, PEN4 and PEN5^62,63^. Of these, PEN2, PEN3, PEN4 can be found in *L. vannamei*^62^. Penaeidins are strongly effective against Gram-positive bacteria, but not Gram-negative bacteria^62^. Lysozymes are a bacteriolytic enzyme that exists in both prokaryotes and eukaryotes and can fight against both Gram-negative and -positive bacteria^64^. In addition to destroying the viral envelope, polyamine CQDs may damage the intestinal bacteria simultaneously. Lysozyme is an antimicrobial protein, and it hydrolyses bacterial cell wall peptidoglycan (PG) that can activate pattern recognition receptors^65^. Therefore, the degradation of bacteria may modulate the host immune response. Although the expression levels of immune genes did not increase with increasing dose, polyamine CQDs did have immunostimulatory effects in shrimp. The relationship between the expression levels of immune genes and the dose of polyamine CQDs needs to be explored in the future.

The composition of most foods contains carbon, hydrogen, oxygen and nitrogen, and many nanoparticles are produced during food manufacturing process. Carbon quantum dots (CQDs) can be easily produced via heating reaction and are found in most food, like Coca-Cola and Pepsi-Cola^66^. The mice fed with high dose Coca-Cola and Pepsi-Cola CQDs do not appear obvious organ damage, and CQDs could be digested in the small intestine or cleared through the liver. Besides, the raw materials of polyamine are natural substances and exist in nearly all organisms^21^. In our previous study, polyamine CQDs do not show obvious cytotoxicity and induce hemolysis reaction with the concentration up to 100 μg/mL. Moreover, polyamine CQDs could be used to treat eye-related bacterial infections in rabbits without any side effects^27^. Therefore, low dose polyamine CQDs containing feed are safe for shrimp.

Many studies have indicated that nanoparticles can replace antibiotics to kill pathogens. Among them, carbon nanoparticles have been verified to be safer than metal nanoparticles by our team members^27^ and other research teams^67,68^, and this study is the first to explore the application of carbon nanoparticles to prevent aquatic diseases. Polyamine CQD materials are inexpensive and their preparation is easy. In this experiment, polyamine CQDs inhibited virus infection and also improved shrimp innate immunity. Therefore, polyamine CQDs may have the ability to inhibit other pathogens, but this is speculative, and requires more experimentation in the future. However, this study has demonstrated that polyamine CQDs have great potential to use as feed additive to against WSSV in shrimp aquaculture.

## References

[1] Nakano H (1994). Mass mortalities of cultured kuruma shrimp, *Penaeus japonicus*, in japan in 1993: Epizootiological survey and infection trials. Fish Pathol..

[2] Wang YC, Lo CF, Chang PS, Kou GH (1998). Experimental infection of white spot baculovirus in some cultured and wild decapods in Taiwan. Aquaculture.

[3] Vaseeharan B, Jayakumar R, Ramasamy P (2003). PCR-based detection of white spot syndrome virus in cultured and captured crustaceans in india. Lett. Appl. Microbiol..

[4] Sanchez-Paz A (2010). White spot syndrome virus: An overview on an emergent concern. Vet. Res..

[5] Leu JH (2009). Whispovirus. Curr. Top. Microbiol. Immunol..

[6] Chou HY, Huang CY, Wang CH, Chiang HC, Lo C (1995). Pathogenicity of a baculovirus infection causing white spot syndrome in cultured penaeid shrimp in Taiwan. Dis. Aquat. Org..

[7] Galaviz-Silva L, Molina-Garza ZJ, Alcocer-Gonzalez JM, Rosales-Encinas JL, Ibarra-Gamez C (2004). White spot syndrome virus genetic variants detected in Mexico by a new multiplex PCR method. Aquaculture.

[8] Hasson KW, Fan Y, Reisinger T, Venuti J, Varner PW (2006). White-spot syndrome virus (WSSV) introduction into the Gulf of Mexico and Texas freshwater systems through imported, frozen bait-shrimp. Dis. Aquat. Organ..

[9] Escobedo-Bonilla CM (2008). A review on the morphology, molecular characterization, morphogenesis and pathogenesis of white spot syndrome virus. J. Fish Dis..

[10] Di Leonardo VA, Bonnichon V, Roch P, Parrinello N, Bonami JR (2005). Comparative wssv infection routes in the shrimp genera *Marsupenaeus* and *Palaemon*. J. Fish Dis..

[11] Wang YG, Hassan MD, Shariff M, Zamri SM, Chen X (1999). Histopathology and cytopathology of white spot syndrome virus (WSSV) in cultured *Penaeus monodon* from peninsular Malaysia with emphasis on pathogenesis and the mechanism of white spot formation. Dis. Aquat. Organ..

[12] Wongteerasupaya C (1996). DNA fragment of *Penaeus monodon* baculovirus PmNOBII gives positive *in situ* hybridization with white-spot viral infections in six penaeid shrimp species. Aquaculture.

[13] Chen LL, Lu LC, Wu WJ, Lo CF, Huang WP (2007). White spot syndrome virus envelope protein VP53A interacts with *Penaeus monodon* chitin-binding protein (PmCBP). Dis. Aquat. Organ..

[14] Huang HT, Chan HL, Shih TY, Chen LL (2015). A study of the role of glucose transporter 1 (Glut1) in white spot syndrome virus (WSSV) infection. Fish Shellfish Immunol..

[15] Chaivisuthangkura P (2006). Development of a polyclonal antibody specific to VP19 envelope protein of white spot syndrome virus (WSSV) using a recombinant protein preparation. J. Virol. Methods.

[16] Ha YM (2008). Vaccination of shrimp (*Penaeus chinensis*) against white spot syndrome virus (WSSV). J. Microbiol. Biotech..

[17] Tang X, Wu J, Sivaraman J, Hew CL (2007). Crystal structures of major envelope proteins VP26 and VP28 from white spot syndrome virus shed light on their evolutionary relationship. J. Virol..

[18] van Hulten MC, Witteveldt J, Snippe M, Vlak JM (2001). White spot syndrome virus envelope protein VP28 is involved in the systemic infection of shrimp. Virology.

[19] Witteveldt J, Vlak JM, van Hulten MC (2004). Protection of *Penaeus monodon* against white spot syndrome virus using a WSSV subunit vaccine. Fish Shellfish Immunol..

[20] Lee YJ, Chen LL (2017). WSSV envelope protein VP51B links structural protein complexes and may mediate virus infection. J. Fish Dis..

[21] Lim SY, Shen W, Gao ZQ (2015). Carbon quantum dots and their applications. Chem. Soc. Rev..

[22] Meziani MJ (2016). Visible-light-activated bactericidal functions of carbon “Quantum” dots. Acs Appl Mater Inter.

[23] Kuo WS (2017). Graphene quantum dots with nitrogen-doped content dependence for highly efficient dual- modality photodynamic antimicrobial therapy and bioimaging. Biomaterials.

[24] Sun H, Gao N, Dong K, Ren J, Qu X (2014). Graphene quantum dots-band-aids used for wound disinfection. ACS Nano.

[25] Harroun SG, Lai JY, Huang CC, Tsai SK, Lin HJ (2017). Reborn from the ashes: Turning organic molecules to antimicrobial carbon quantum dots. ACS Infect. Dis..

[26] Li YJ (2016). Synthesis of self-assembled spermidine-carbon quantum dots effective against multidrug-resistant bacteria. Adv. Healthc. Mater..

[27] Jian HJ (2017). Super-cationic carbon quantum dots synthesized from spermidine as an eye drop formulation for topical treatment of bacterial keratitis. ACS Nano.

[28] Galdiero S (2011). Silver nanoparticles as potential antiviral agents. Molecules.

[29] Lu L (2008). Silver nanoparticles inhibit hepatitis B virus replication. Antivir. Ther..

[30] Orlowski, P. *et al*. Antiviral activity of tannic acid modified silver nanoparticles: potential to activate immune response in herpes genitalis. *Viruses-Basel* **10**(10) (2018). 10.3390/v10100524 PMC6213294 30261662

[31] Tassanakajon A (2013). Innate immune system of shrimp. Fish Shellfish Immun..

[32] Jiravanichpaisal P, Lee BL, Soderhall K (2006). Cell-mediated immunity in arthropods: hematopoiesis, coagulation, melanization and opsonization. Immunobiology.

[33] Sivaramasamy E, Zhiwei W, Li F, Xiang JJJNN (2016). Enhancement of vibriosis resistance in *Litopenaeus vannamei* by supplementation of biomastered silver nanoparticles by *Bacillus subtilis*. J. Nanomed. Nanotechnol..

[34] Ishwarya R (2018). *Sargassum wightii*-synthesized ZnO nanoparticles - from antibacterial and insecticidal activity to immunostimulatory effects on the green tiger shrimp *Penaeus semisulcatus*. J. Photoch. Photobio. B.

[35] Ochoa-Meza AR (2019). Silver nanoparticles enhance survival of white spot syndrome virus infected *Penaeus vannamei* shrimps by activation of its immunological system. Fish Shellfish Immun..

[36] Kandasamy K, Alikunhi NM, Manickaswami G, Nabikhan A, Ayyavu G (2013). Synthesis of silver nanoparticles by coastal plant *Prosopis chilensis* (L.) and their efficacy in controlling vibriosis in shrimp *Penaeus monodon*. Appl. Nanosci..

[37] Tello-Olea M (2019). Gold nanoparticles (AuNP) exert immunostimulatory and protective effects in shrimp (*Litopenaeus vannamei*) against *Vibrio parahaemolyticus*. Fish Shellfish Immunol.

[38] Zhou Q, Qi YP, Yang F (2007). Application of spectrophotometry to evaluate the concentration of purified white spot syndrome virus. Journal of Virological Methods.

[39] Wang KCHC (2010). RNAi knock-down of the *Litopenaeus vannamei Toll* gene (*LvToll*) significantly increases mortality and reduces bacterial clearance after challenge with *Vibrio harveyi*. Dev. Comp. Immunol..

[40] Livak KJ, Schmittgen TD (2001). Analysis of relative gene expression data using real-time quantitative PCR and the 2 -ΔΔCT method. J. Methods.

[41] Corteel M (2009). Molt stage and cuticle damage influence white spot syndrome virus immersion infection in penaeid shrimp. Vet. Microbiol..

[42] Tsai MF (2000). Transcriptional analysis of the ribonucleotide reductase genes of shrimp white spot syndrome virus. Virology.

[43] Dhar AK, Cowley JA, Hasson KW, Walker PJ (2004). Genomic organization, biology, and diagnosis of Taura syndrome virus and yellowhead virus of penaeid shrimp. Adv. Virus Res..

[44] Elechiguerra JL (2005). Interaction of silver nanoparticles with HIV-1. J Nanobiotechnology.

[45] Sun RWY (2005). Silver nanoparticles fabricated in Hepes buffer exhibit cytoprotective activities toward HIV-1 infected cells. Chem Commun.

[46] Márquez JCM (2018). Silver nanoparticles applications (AgNPs) in aquaculture. *Int*. J. Fish. Aquat. Stud..

[47] Rafiei S, Rezatofighi SE, Roayaei Ardakani M, Rastegarzadeh S (2015). Gold nanoparticles impair foot-and-mouth disease virus replication. IEEE Trans Nanobioscience..

[48] Sang X (2007). Photocatalytic inactivation of diarrheal viruses by visible-light-catalytic titanium dioxide. Clin. Lab..

[49] Lauster D (2017). Multivalent peptide-nanoparticle conjugates for influenza-virus inhibition. Angew. Chem. Int. Ed. Engl..

[50] Barmo C (2013). *In vivo* effects of n-TiO_2_ on digestive gland and immune function of the marine bivalve *Mytilus galloprovincialis*. Aquat. Toxicol..

[51] Canesi L (2012). *Bivalve molluscs* as a unique target group for nanoparticle toxicity. Mar. Environ. Res..

[52] Ciacci C (2012). Immunomodulation by different types of N-oxides in the hemocytes of the marine bivalve *Mytilus galloprovincialis*. Plos One.

[53] Bigorgne E (2011). Ecotoxicological assessment of TiO_2_ byproducts on the earthworm *Eisenia fetida*. Environ. Pollut..

[54] Morales-Covarrubias MS, Garcia-Aguilar N, Bolan-Mejia MD, Puello-Cruz AC (2016). Evaluation of medicinal plants and colloidal silver efficiency against *Vibrio parahaemolyticus* infection in *Litopenaeus vannamei* cultured at low salinity. Dis. Aquat. Organ..

[55] Wang XW, Wang JX (2013). Pattern recognition receptors acting in innate immune system of shrimp against pathogen infections. Fish Shellfish Immunol..

[56] Maningas MB, Kondo H, Hirono I (2013). Molecular mechanisms of the shrimp clotting system. Fish Shellfish Immunol..

[57] Maningas MBB, Kondo H, Hirono I, Saito-Taki T, Aoki T (2008). Essential function of transglutaminase and clotting protein in shrimp immunity. Mol Immunol.

[58] Liu CH, Tseng MC, Cheng W (2007). Identification and cloning of the antioxidant enzyme, glutathione peroxidase, of white shrimp, *Litopenaeus vannamei*, and its expression following *Vibrio alginolyticus* infection. Fish Shellfish Immunol.

[59] Harman D (1991). The aging process: Major risk factor for disease and death. Proc Natl Acad Sci USA.

[60] Jiang HS (2015). A new group of anti-lipopolysaccharide factors from *Marsupenaeus japonicus* functions in antibacterial response. Dev. Comp. Immunol..

[61] Banerjee D, Maiti B, Girisha SK, Venugopal MN, Karunasagar I (2015). A crustin isoform from black tiger shrimp, *Penaeus monodon* exhibits broad spectrum anti-bacterial activity. Aquacult. Rep..

[62] Cuthbertson BJ (2008). Diversity in penaeidin antimicrobial peptide form and function. Dev. Comp. Immunol..

[63] Gueguen Y (2006). PenBase, the shrimp antimicrobial peptide penaeidin database: Sequence-based classification and recommended nomenclature. Dev Comp. Immunol..

[64] Zhao XF, Wang JX (2008). The antimicrobial peptides of the immune response of shrimp. Inv. Survl. J..

[65] Simser JA, Macaluso KR, Mulenga A, Azad AF (2004). Immune-responsive lysozymes from hemocytes of the American dog tick, *Dermacentor variabilis* and an embryonic cell line of the rocky mountain wood tick, *D. andersoni*. Insect. Biochem. Mol. Biol..

[66] Li S, Jiang C, Wang H, Cong S, Tan M (2018). Fluorescent nanoparticles present in Coca-Cola and Pepsi-Cola: Physiochemical properties, cytotoxicity, biodistribution and digestion studies. Nanotoxicology.

[67] Sun HJ, Gao N, Dong K, Ren JS, Qu XG (2014). Graphene quantum dots-band-aids used for wound disinfection. Acs Nano.

[68] Anand A (2019). Graphene oxide and carbon dots as broad-spectrum antimicrobial agents - a minireview. Nanoscale Horiz.

